# Accuracy of DentalMonitoring’s artificial intelligence in detecting common orthodontic braces treatment related emergencies

**DOI:** 10.1038/s41598-026-37329-w

**Published:** 2026-01-29

**Authors:** Julie Fahl McCray, Logan Smith, Dylan Handlin, William Dabney, Courtney Mietz, Rayan Skafi, Mohammed H. Elnagar

**Affiliations:** 1 Department of Orthodontics, Center of Advanced Dental Education, Saint Louis University, 3320 Rutger St., St. Louis, MO 63104 USA; 2 Private Practice, 304 Browns Hill Court, Midlothian, VA 23114 USA; 3 DentalMonitoring, Paris, France; 4 Department of Orthodontics (M/C 841), College of Dentistry, University of Illinois Chicago, 801 S. Paulina Street, RM 131, Chicago, IL 60612-7211 USA; 5 Department of Orthodontics, Faculty of Dentistry, Tanta University, Tanta, Egypt

**Keywords:** Diseases, Health care, Medical research

## Abstract

This study aimed to evaluate the diagnostic performance of DentalMonitoring™ (DM) (Dental Monitoring SAS, Paris, France), an FDA-cleared AI-powered orthodontic remote monitoring software, in detecting three common orthodontic appliance issues: bracket debonding, open self-ligating clips, and tie loss. Datasets from 1,014 US-based patients were analyzed. Each DM image set was assessed by the AI algorithm and independently reviewed by a panel of three orthodontic experts, whose consensus served as the reference standard. A total of 659 evaluations were included for bracket debonding, 647 for self-ligating clip status, and 653 for tie presence. Sensitivity and specificity along with their 95% confidence intervals were calculated using a two-level evaluation basis (positive vs. negative) across the three clinical parameters. DM’s AI demonstrated high diagnostic performance, with sensitivity of 98.4% for bracket debonding, 91.1% for open self-ligating clips, and 93.3% for tie loss. Corresponding specificity values were 99.6%, 88.3%, and 96.5%, respectively. Current results indicate that DM’s AI analysis system has high accuracy in detecting bracket debonding, open self-ligating clips, and tie loss. DM can help significantly reduce the rate of these undetected clinical incidents, providing a better approach to managing emergencies and maintaining clinical control in orthodontic treatment.

## Introduction

 In the realm of orthodontic treatment, the popularity of clear aligners has been remarkable. Yet, despite this burgeoning trend, conventional fixed braces persist as the most widely adopted and the most comprehensive approach used for treating most malocclusions^1,2^. The three primary factors that underpin this enduring preference are the comparative affordability of conventional bracket systems, the substantiated superior occlusal outcomes they offer, as corroborated by recent meta-analyses, and the high rate of refinements associated with clear aligner treatments that undermine their impact on efficiency and predictability^3–5^.

To meet the ever-evolving expectations of patients and practitioners alike, fixed appliances have evolved significantly. Manufacturers today claim various advantages such as reduced friction, decreased treatment time, longer treatment intervals with fewer appointments, and less patient discomfort, however, sufficient evidence still needs to be provided^6–8^.

While these technological advancements have greatly enhanced the efficiency and aesthetics of fixed orthodontic appliances, they have not eliminated all clinical limitations. Studies have underscored the elevated incidence of orthodontic-related emergencies associated with all kinds of brackets, deemed more common than treatments with clear aligners^9^. Broken or detached appliances, loosening of molar bands, and mucosa injury due to a displaced or protruded wire were the most reported emergencies^10^. The existing literature doesn’t explore the rates of other clinical incidents such as degrading or loss of ties with conventional brackets and open clips with self-ligating bracket appliances.

In many cases, these issues go unnoticed by patients, who may be unaware of detached components or even accidental ingestion, particularly among children and adolescents^11–13^. Limited visibility into such incidents outside the clinical setting can also result in treatment inefficiencies, unexpected repairs, and extended treatment times, all of which may negatively affect patient satisfaction^14^.

Importantly, no studies to date have evaluated whether artificial intelligence (AI)–based systems can accurately detect or report these minor bracket-related incidents, representing a critical gap in current orthodontic research.

**Fig. 1:**
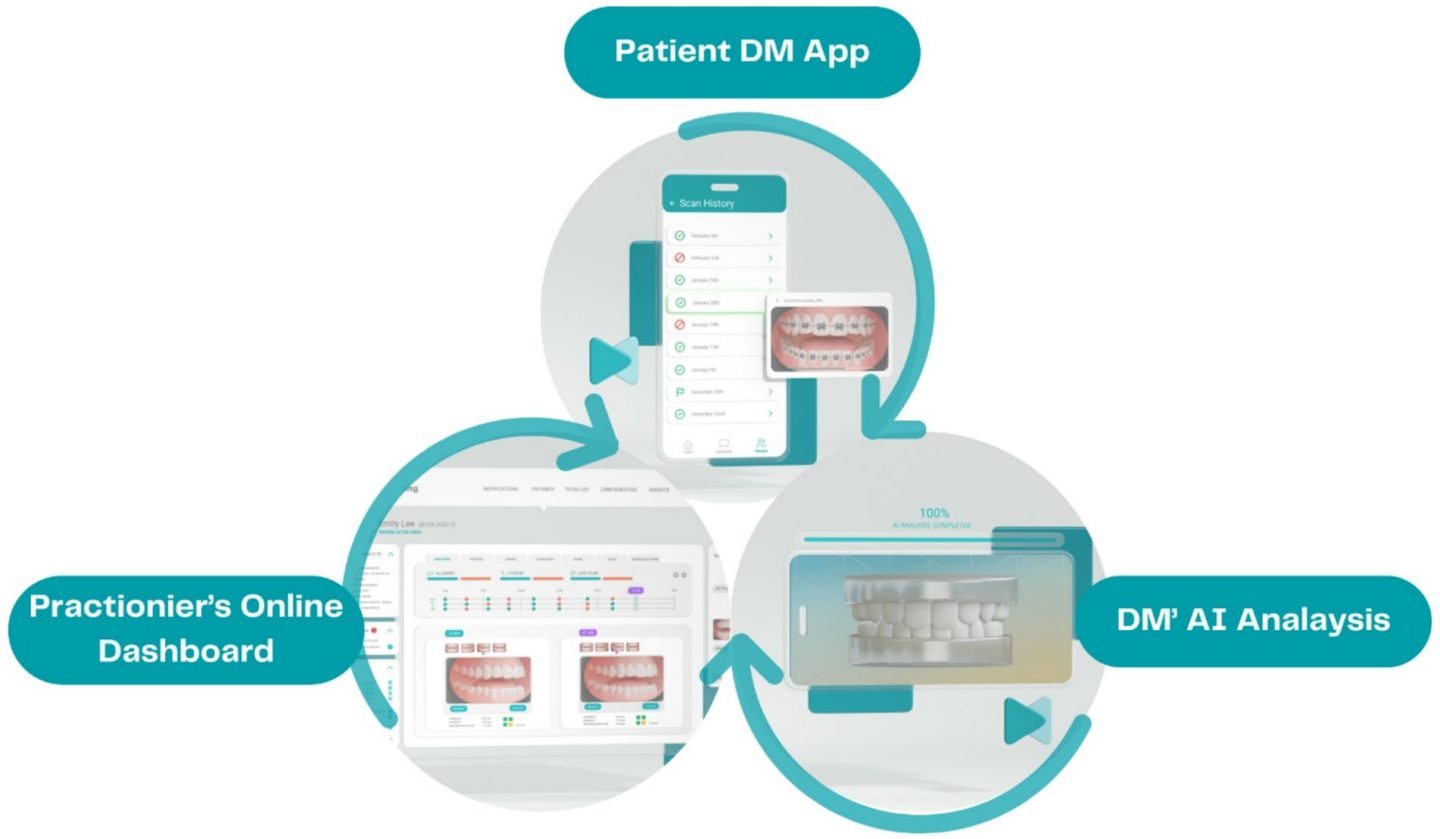
DM’s 3 interconnected modules.

**Fig. 2:**
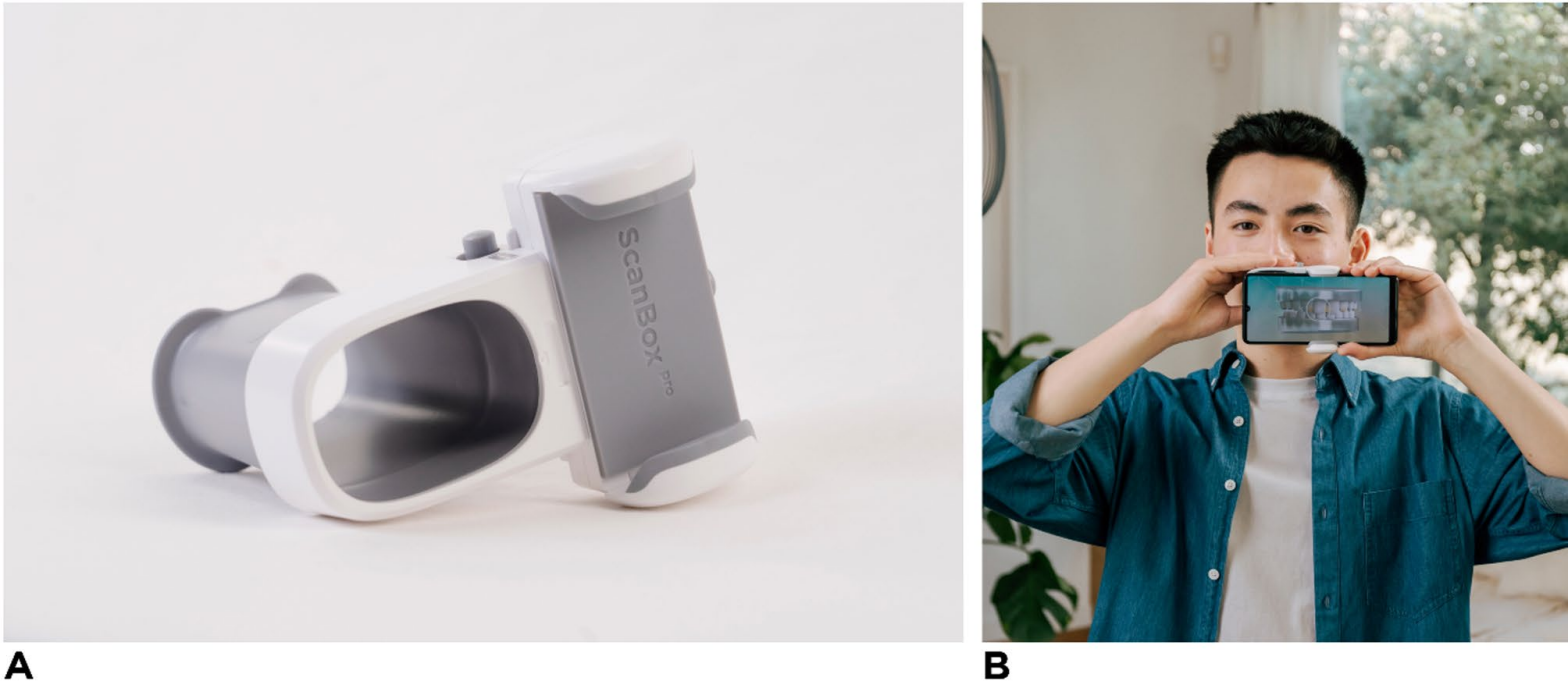
(**A**) The DentalMonitoring proprietary hardware. (**B**) The scanning process.

The aim of this study was to evaluate the accuracy of DentalMonitoring™ (DM) (Dental Monitoring SAS, Paris, France), a novel AI-powered remote monitoring software recently approved by the FDA, in detecting specific clinical incidents including bracket debonding, open self-ligating clips, and tie loss. DM consists of three interconnected modules: a smartphone application for patients, a cloud platform hosting patented AI algorithms, and a web-based doctor dashboard for orthodontists to monitor treatment progress (Fig. 1). This system enables asynchronous interaction via the app while allowing orthodontists to regularly track treatment progress through picture sets provided by DM scans, which patients perform at home using the DM proprietary hardware (Fig. 2). The DM software employs AI technology to detect clinical incidents related to orthodontic appliances, oral hygiene issues, and tooth movement.

## Materials and methods

### Study design and rationale

The study was conducted following the ethical principles outlined in the Declaration of Helsinki and adhered strictly to applicable data protection regulations, including Regulation (EU) 2017/745 and ISO 14155:2020. An exemption from the WCG Institutional Review Board (IRB) was granted under 45 CFR § 46.104(d)(4), **WCG IRB NO 1–1461410-1**. Because this study was a retrospective study the WCG IRP granted exemption/wavier for a Informed Consent form (Informed Consent to Participate declarations: not applicable). The person/model in Fig. 2 holding the DM scanbox has signed a consent form for the purpose of publication.

This retrospective, multicenter qualitative comparative study included 1,014 patients selected from a large dataset extracted from the DM database, representing multiple orthodontic practices across the US. The dataset was specifically compiled as part of an FDA regulatory submission for approval of the DM system as Software as a Medical Device (SaMD), thus requiring a substantial sample size to demonstrate safety, effectiveness, and generalizability. All cases analyzed were real-world DM cases extracted from the production database, in which DM scans had already been automatically processed and classified by the relevant AI detection algorithm (e.g., bracket loss detected or no issue detected). To ensure balanced and statistically robust datasets, cases were randomly selected according to algorithm outputs for each incident type and predefined statistical thresholds:

- *Bracket debonding*: ≥300 patients; ≥660 total tooth-level results (130–160 positive, 530–580 negative).
- *Tie loss*: ≥300 patients (≥ 60 positive, ≥ 240 negative).
- *Open self-ligating clip*: ≥300 patients (≥ 60 positive, ≥ 240 negative).

These criteria ensured sufficient variability and representation of both positive and negative cases for reliable sensitivity and specificity estimation.

The patients’ inclusion criteria were: Adolescents and adults aged 13 years and older undergoing treatment with fixed appliances, including metallic and ceramic brackets in both conventional and self-ligating systems. As cases were randomly sampled across multiple stages of treatment, the dataset naturally included a broad variety of orthodontic archwires. The patients’ exclusion criteria were: Patients with at least one primary tooth, and patients younger than 13 years old. With regard to the picture sets generated from the DM scans, the inclusion criteria were: De-identified DM picture sets, picture set acquired using the DM app (on a phone with at least Android 6 and up, or iOS 11 and up), a DM Cheek Retractor and a DM ScanBox and lastly a DM picture set with at least eight images processable by DM respecting the following reparation: At least three closed-view images (left, right, and front), at least three open-view images (left, right, and front), and two occlusal-view images (up and down), although the latter were not mandatory.

To ensure that the analysis covers all the teeth of a patient’s dentition, it was verified that each tooth number represented at least 1% of the total number analyzed. The breakdown analysis per tooth for each parameter is detailed in Appendix A.

All teeth were represented for the bracket debonding parameter. For the tie loss parameter, all the teeth are represented in the analysis except teeth 16, 26, 46, 17, 27, 37 and 47, which are molars where ties are typically not placed. Finally, for the self-ligating parameter, all the teeth were represented in the analysis except teeth 17, 27, 37, and 47.

**Fig. 3:**
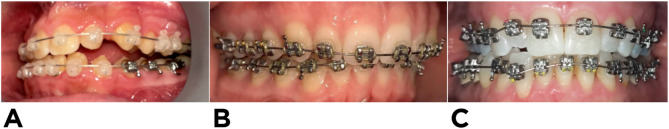
(**A**) Bracket Debonding UL4, (**B**) Loss of Tie LR5, (**C**) Open Clip UR1.

The study aimed to evaluate DM’s performances for detecting bracket debdondings, loss of ties and open self-ligating clips. Some examples are illustrated in Fig. 3. Fulfillment of the objective was assessed per clinical parameter and not globally.

Prior to the initiation of the study, comprehensive training was provided by DM’s Clinical Affairs team to the participating experts. The objective of this training was to ensure experts were thoroughly familiar with the investigation procedures, study definitions, labeling tools, and data-entry protocols. To minimize potential bias, the Clinical Affairs team’s involvement was strictly limited to procedural training and troubleshooting prior to the formal initiation of the study. They were not involved in patient recruitment, image acquisition, or data analysis during the study.Experts underwent a familiarization period using representative mock cases, independent of the actual study dataset, to validate their adherence to the protocol and ensure consistency in interpretation. This phase also served as a calibration exercise aimed at aligning evaluation criteria across experts and minimizing inter-rater variability. Each expert working on the study was provided with a dedicated tool to perform patient assessments. In addition, all experts performed their evaluations using screens standardized for size and resolution to ensure uniform assessment conditions. Supplemental training on the established reading scale was also provided to the expert panel, as this method is recommended and validated within the radiology field^15^.

**Fig. 4:**
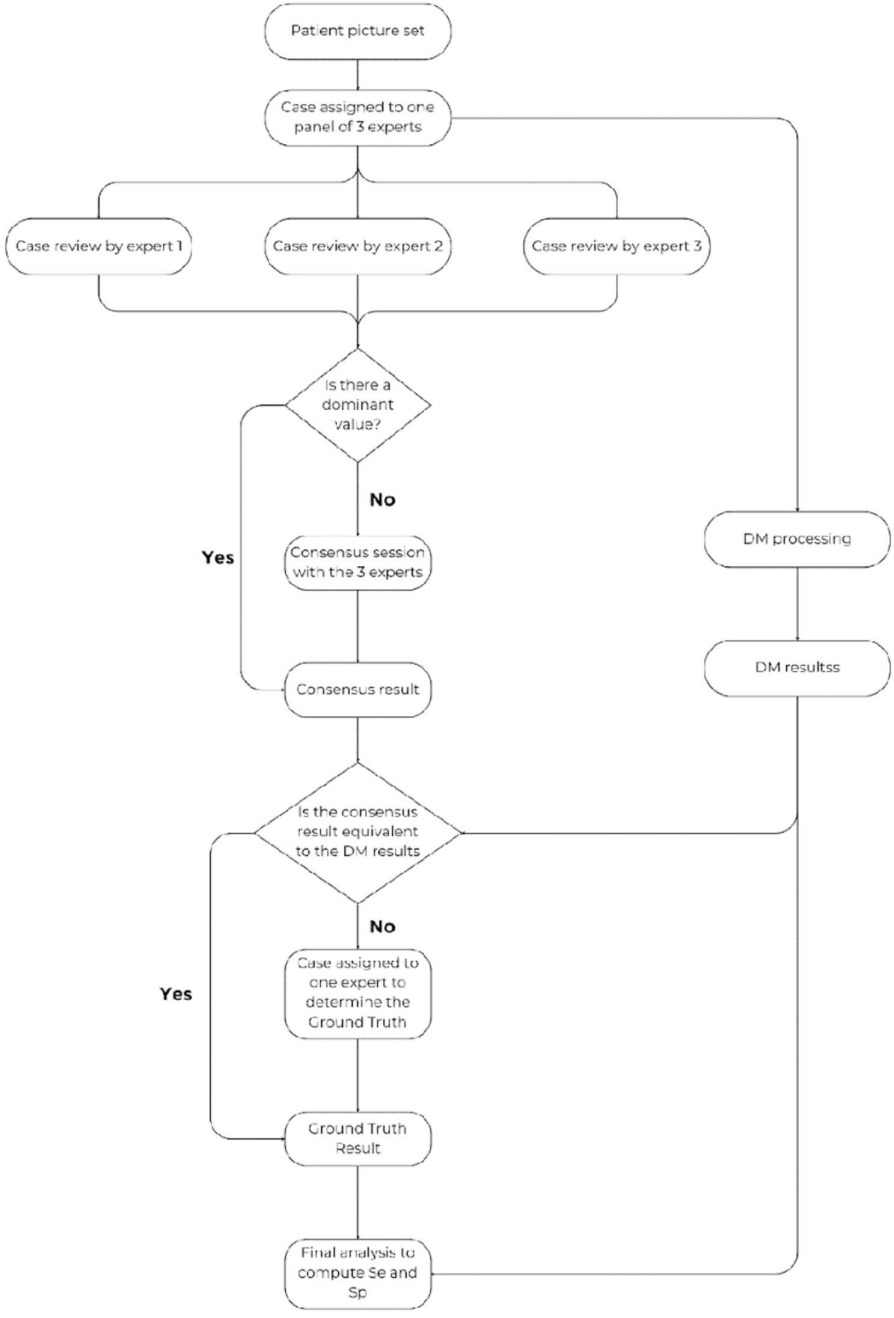
The detailed process of the reference method.

Each selected DM picture set, generated from a single DM scan and comprising a minimum of eight intraoral images, was processed by DM to produce the AI-generated result. The same image set was independently reviewed by a panel of three orthodontic experts to establish a reference (ground truth) result. For each tooth and clinical parameter, the following consensus methodology was applied (Fig. 4):

- If all three experts provided identical assessments, that value was recorded as the panel’s final result.
- If two experts provided matching assessments, this majority value was recorded as the final result.
- If all three assessments differed (no consensus or majority), the case was re-evaluated through a group discussion to reach a consensus, which was then recorded as the final panel result.

In situations where the DM result and the panel consensus disagreed, the case was flagged as potentially ambiguous or difficult to interpret (e.g., limited image clarity, partial bracket detachment, or borderline findings). To minimize the potential effect of human fatigue or perceptual bias in these challenging cases, an external expert, independent from the initial panel, was asked to review the complete image set along with the DM result and the experts’ consensus. This review aimed solely to adjudicate the disagreement and define the **final Ground Truth**, which served as the reference standard for the statistical analysis. The external expert was provided with:

- The initial answer given by the consensus of experts = Consensus result.
- The answer given by DM = The DM result.
- Definition of the clinical parameter being evaluated with illustrating examples to increase objectivity.
- Full picture set of the patient.

### Statistical analysis

The sensitivity and specificity, with their 95% confidence intervals, were calculated using a Generalized Estimating Equations (GEE) model. This approach accounts for correlations between multiple teeth from the same patient, ensuring robust estimates. The model is particularly suited for this study, where each patient contributes multiple results due to the inclusion of several teeth.

To evaluate the diagnostic performance of the candidate method, results were classified in comparison to the ground truth using a standard 2 × 2 contingency framework. A result was considered a true positive (TP) when the method correctly identified a positive case, and a true negative (TN) when it correctly identified a negative case. A false positive (FP) indicated an incorrect identification of a negative case as positive, while a false negative (FN) referred to a missed positive case.

Based on this classification, sensitivity was calculated as TP/(TP + FN), representing the method’s ability to correctly detect true positive findings. Specificity was calculated as TN/(FP + TN), reflecting the method’s accuracy in identifying true negatives. These metrics were used to quantify the reliability of the system in detecting clinical events.

## Results

Descriptive statistics for all recruited subjects who completed the study were performed for age (years), location and treatment type (Table 1).

**Table 1:** Demographic description of the population included in the statistical analysis for bracket debonding, loss of tie and self-ligating clip.

Characteristic	Bracket debonding	Loss of tie	Self-ligating clip
	*n* (%)	*n* (%)	*n* (%)
Age group			
12–22 years of age	270 (71.2%)	240 (77.4%)	217 (66.8%)
≥ 22 years of age	107 (28.2%)	66 (21.3%)	103 (31.7%)
≤ 12 years old	2 (0.5%)	4 (1.3%)	5 (1.5%)
Location			
Central	291 (76.8%)	255 (82.3%)	225 (69.2%)
East Coast	60 (15.8%)	39 (12.6%)	69 (21.2%)
West Coast	16 (4.2%)	10 (3.2%)	16 (4.9%)
Out of US	1 (0.3%)	3 (1.0%)	12 (3.7%)
Western	11 (2.9%)	3 (1.0%)	3 (0.9%)
Treatment type			
Brackets - Ceramic	10 (2.6%)	13 (4.2%)	5 (1.5%)
Brackets - Self-ligating	171 (45.1%)	5 (1.6%)	262 (80.6%)
Brackets - Traditional	150 (39.6%)	267 (86.1%)	21 (6.5%)
Mixed treatment	48 (12.6%)	25 (8.1%)	37 (11.4%)

The final comparison between DM and Ground Truth for the 3 clinical parameters is presented in Tables 2, 3 and 4. The Sensitivity and Specificity along with the 95% CI of the product for the three clinical parameters were computed using the GEE model to take the multiplicity of results per patient into account (Table 5).

The DM system demonstrated high diagnostic accuracy across all incident types when compared with the Ground Truth assessments (Tables 2, 3, 4 and 5). A total of 659 bracket debonding, 653 tie loss, and 647 self-ligating clip assessments were analyzed.

For bracket debonding, the system achieved a sensitivity of 98.4% (95% CI: 93.8–99.6%) and a specificity of 99.6% (95% CI: 98.5–99.9%), indicating near-perfect detection performance.

For tie loss, sensitivity reached 93.3% (95% CI: 85.7–97.0%) with a specificity of 96.5% (95% CI: 94.0–98.0%), while for self-ligating clip incidents, sensitivity and specificity were 91.1% (95% CI: 82.5–95.7%) and 88.3% (95% CI: 84.1–91.5%), respectively.

These results demonstrate strong agreement between DM and expert evaluations, with particularly high accuracy for bracket debonding and tie loss detection.

**Table 2:** Final comparison between DentalMonitoring and ground truth for bracket debonding.

		Ground truth		
		Positive	Negative	Total
DM result	Positive	127	2	129
	Negative	2	528	530
	Total	129	530	659

**Table 3:** Final comparison between DentalMonitoring and ground truth for tie loss.

		Ground truth		
		Positive	Negative	Total
DM result	Positive	111	15	126
	Negative	7	520	527
	Total	118	535	653

**Table 4:** Final comparison between DentalMonitoring and ground truth for self-ligating clip.

		Ground truth		
		Positive	Negative	Total
DM result	Positive	71	48	119
	Negative	7	521	528
	Total	78	569	647

**Table 5:** Final study results for bracket debonding, Self-ligating clip, and tie loss.

Sensitivity	Bracket debonding	Self-ligating clip	Tie loss
	98.4%	91.1%	93.3%
Sensitivity 95% CI	[93.8%, 99.6%]	[82.5%, 95.7%]	[85.7%, 97.0%]
Specificity	99.6%	88.3%	96.5%
Specificity 95% CI	[98.5%, 99.9%]	[84.1%, 91.5%]	[94.0%, 98.0%]

## Discussion

This unique study aimed to explore the reliability of Artificial Intelligence Driven Remote Monitoring (AIDRM) in detecting 3 common fixed orthodontic-related emergencies in real-time: Bracket debonding, open self-ligating clip, and tie loss. The study protocol ensured the inclusion of a variety of appliance systems, including ceramic, traditional metal, and self-ligating brackets, while also striving to cover as many teeth as possible in an equitable manner to provide a more representative sample of reality. The results demonstrated very high sensitivity and specificity for all three parameters. This finding indicates that DM can accurately identify these issues following a patient scan, with a very low margin of error for false positives and false negatives. For example, in the case of bracket debonding incidents, which are considered the most commonly reported emergencies in braces treatments, the system underreported them in less than 2% of cases. For the other 2 parameters, the margin of error was less than 9%. This low margin of error is deemed acceptable when considering the risk-benefit ratio. It is important to note that these results are based on analyzing a single picture set per parameter per patient, which does not represent the actual usage of DM that involves regular repeated scans. During typical fixed orthodontic treatment, patients are usually advised to scan weekly or bi-weekly throughout the treatment duration. This means that any issues missed in a specific picture set due to insufficient visibility or low image quality have an increased probability of detection with each subsequent scan (given that the patient is respecting the scanning guidelines). Additionally, as mentioned previously, DM is not intended to replace the orthodontist’s supervision and clinical decision-making process but rather to provide assistance, which can significantly decrease the rate of undetected clinical incidents, especially between prolonged appointment intervals.

Exploring the literature indicates that remote monitoring has never gained significant popularity and attention in orthodontics until the COVID-19 pandemic^16–21^. During that period, orthodontists leveraged various basic digital communication technologies such as WhatsApp^®^ Messenger and Zoom^®^to ensure the continuity of treatment, communicate with patients, and manage emergencies. This approach proved valuable for professionals and patients facing orthodontic emergencies during that time, especially with fixed orthodontic appliances^22,23^. This experience also taught us that more attention should be given to teleorthodontics as this approach is considered an appropriate solution and addition even in normal times to better detect and manage emergencies, while reducing time and money spent, without a decrease in orthodontic quality^24,25^. However, this approach can be burdensome, time-consuming, and inefficient if implemented alone, hence the role of AI assistance to make it scalable and efficient^26^.

We note several limitations in this study. First, the retrospective design limited control over image acquisition conditions, which may have introduced variability in scan quality. Although expert consensus was used to establish the reference standard, the absence of chairside clinical confirmation may limit its objectivity. Additionally, the analysis was based on a single DM picture set per patient, without repeated scans or longitudinal follow-up. As such, the reproducibility of DM’s AI-generated results over time or across multiple assessments was not evaluated and warrants further investigation in prospective settings.

In this endeavor to strive towards an “AI-driven orthodontic utopia,” the pertinent issue that needs to be addressed is setting high standards for algorithms to ensure safety and consider the legal implications of AI-based clinical decisions^27,28^. Future research endeavors should meticulously assess the algorithms’ efficacy while examining the ramifications of this innovative approach on patient care quality, experience, and treatment efficiency.

## Conclusion

The main findings of this study suggest that DM’s algorithms are highly accurate in detecting the following common fixed orthodontic-treatment-related incidents: Bracket debonding, tie loss, and open self-ligating clips. Orthodontists who remotely monitor their patients’ treatments with DM are instantly notified when these clinical incidents are detected following a patient’s scan and can take swift action accordingly.

Considering the long intervals between appointments and the lack of visibility when the patient is not in the office, Artificial Intelligence Driven Remote Monitoring (AIDRM) can be a valuable tool in aiding orthodontists to better manage fixed orthodontic clinical incidents and emergencies.

## Data Availability

All data generated during this study are included in this published article.

## Appendix A

See Tables 6, 7 and 8.

**Table 6:** Distribution of the teeth included in the analysis for bracket debonding.

Tooth number	Frequency	Tooth number	Frequency
11	3.64%	31	3.49%
12	3.34%	32	3.49%
13	2.28%	33	3.95%
14	5.77%	34	2.88%
15	3.49%	35	3.49%
16	5.61%	36	5.46%
17	2.43%	37	2.58%
21	3.03%	41	3.64%
22	4.55%	42	3.03%
23	3.49%	43	3.49%
24	3.95%	44	3.34%
25	3.03%	45	2.88%
26	2.88%	46	3.49%
27	2.88%	47	4.40%

**Table 7:** Distribution of the teeth included in the analysis for tie loss.

Tooth number	Frequency	Tooth number	Frequency
11	8.1%	32	8.1%
12	7.7%	33	11.6%
13	10.3%	34	9.0%
14	14.2%	35	16.5%
15	14.2%	36	0.3%
21	11.6%	41	10.0%
22	9.4%	42	9.0%
23	7.7%	43	7.7%
24	10.3%	44	8.1%
25	10.3%	45	17.1%
31	10.3%	46	0.0%

**Table 8:** Distribution of the teeth included in the analysis for self-ligating clip.

Tooth number	Frequency	Tooth number	Frequency
11	3.09%	31	4.64%
12	4.17%	32	6.65%
13	4.95%	33	6.03%
14	6.80%	34	3.71%
15	6.80%	35	5.26%
16	0.15%	36	0.15%
21	5.41%	41	6.03%
22	5.10%	42	4.64%
23	4.48%	43	5.10%
24	4.95%	44	2.78%
25	5.10%	45	3.71%
26	0.31%	46	0.0%
